# Association between systemic immune inflammation index, systemic inflammation response index and adult psoriasis: evidence from NHANES

**DOI:** 10.3389/fimmu.2024.1323174

**Published:** 2024-02-13

**Authors:** Rui Ma, Lian Cui, Jiangluyi Cai, Nan Yang, Yuanyuan Wang, Qianyu Chen, Wenjuan Chen, Chen Peng, Hui Qin, Yangfeng Ding, Xin Wang, Qian Yu, Yuling Shi

**Affiliations:** ^1^ Department of Dermatology, Shanghai Skin Disease Hospital, Tongji University School of Medicine, Shanghai, China; ^2^ Institute of Psoriasis, Tongji University School of Medicine, Shanghai, China; ^3^ Department of Dermatology, Shanghai Tenth People’s Hospital, Tongji University School of Medicine, Shanghai, China

**Keywords:** systemic immune-inflammation index, systemic inflammation response index, psoriasis, adults, NHANES

## Abstract

**Background:**

The systemic immune-inflammation index (SII) and systemic inflammation response index (SIRI) are both novel biomarkers and predictors of inflammation. Psoriasis is a skin disease characterized by chronic inflammation. This study aimed to investigate the potential association between SII, SIRI, and adult psoriasis.

**Methods:**

Data of adults aged 20 to 80 years from the National Health and Nutrition Examination Survey (NHANES) (2003–2006, 2009–2014) were utilized. The K-means method was used to group SII and SIRI into low, medium, and high-level clusters. Additionally, SII or SIRI levels were categorized into three groups: low (1^st^-3^rd^ quintiles), medium (4^th^ quintile), and high (5^th^ quintile). The association between SII-SIRI pattern, SII or SIRI individually, and psoriasis was assessed using multivariate logistic regression models. The results were presented as odds ratios (ORs) and confidence intervals (CIs). Restricted cubic spline (RCS) regression, subgroup, and interaction analyses were also conducted to explore the potential non-linear and independent relationships between natural log-transformed SII (lnSII) levels or SIRI levels and psoriasis, respectively.

**Results:**

Of the 18208 adults included in the study, 511 (2.81%) were diagnosed with psoriasis. Compared to the low-level group of the SII-SIRI pattern, participants in the medium-level group had a significantly higher risk for psoriasis (OR = 1.40, 95% CI: 1.09, 1.81, *p*-trend = 0.0031). In the analysis of SII or SIRI individually, both SII and SIRI were found to be positively associated with the risk of psoriasis (high vs. low group OR = 1.52, 95% CI: 1.18, 1.95, *p*-trend = 0.0014; OR = 1.48, 95% CI: 1.12, 1.95, *p*-trend = 0.007, respectively). Non-linear relationships were observed between lnSII/SIRI and psoriasis (both *p*-values for overall < 0.05, *p*-values for nonlinearity < 0.05). The association between SII levels and psoriasis was stronger in females, obese individuals, people with type 2 diabetes, and those without hypercholesterolemia.

**Conclusion:**

We observed positive associations between SII-SIRI pattern, SII, SIRI, and psoriasis among U.S. adults. Further well-designed studies are needed to gain a better understanding of these findings.

## Introduction

Psoriasis is a common chronic inflammatory skin disease that affects over 60 million adults and children worldwide, causing a significant burden on society (1). It is characterized by erythematous and scaly skin lesions that can appear on various parts of the body, accompanied by systemic manifestations (2). The etiology of psoriasis is not fully understood and involves complex interactions between genetic, immune, and environmental factors (3). Psoriasis is currently incurable, but the search for new factors or biomarkers to assess its risk has always attracted extensive attention and is expected to have clinical applications.

Undoubtedly, psoriasis is an inflammatory skin disease in which both systemic and local inflammatory reactions play crucial roles in its onset and progression (1). However, there is limited research available on the relationship between psoriasis and the overall chronic inflammatory status of the body. The systemic immune-inflammation index (SII) and system inflammation response index (SIRI) are integrated and innovative inflammatory biomarker have recently been proposed based on immune cell subpopulation and platelet counts (4, 5). These indices have been widely used in studies to assess the association between chronic inflammatory status and various human diseases, including cancers, metabolic disorders, and inflammatory conditions (6, 7).

The National Health and Nutrition Examination Survey (NHANES) is a comprehensive survey conducted in the United States that utilizes complex, multi-stage, and probability sampling methods to gather nutritional and health information about the population (8). Using the NHANES database, more and more factors related to human health and diseases have been discovered. For instance, recent studies have utilized the NHANES database to investigate the roles of SII and SIRI in various human diseases (9, 10).

Despite the growing body of research, the links between SII/SIRI and psoriasis remain unclear. Therefore, our study aims to explore this relationship using the NHANES database.

## Methods

### Study population

**Figure 1:**
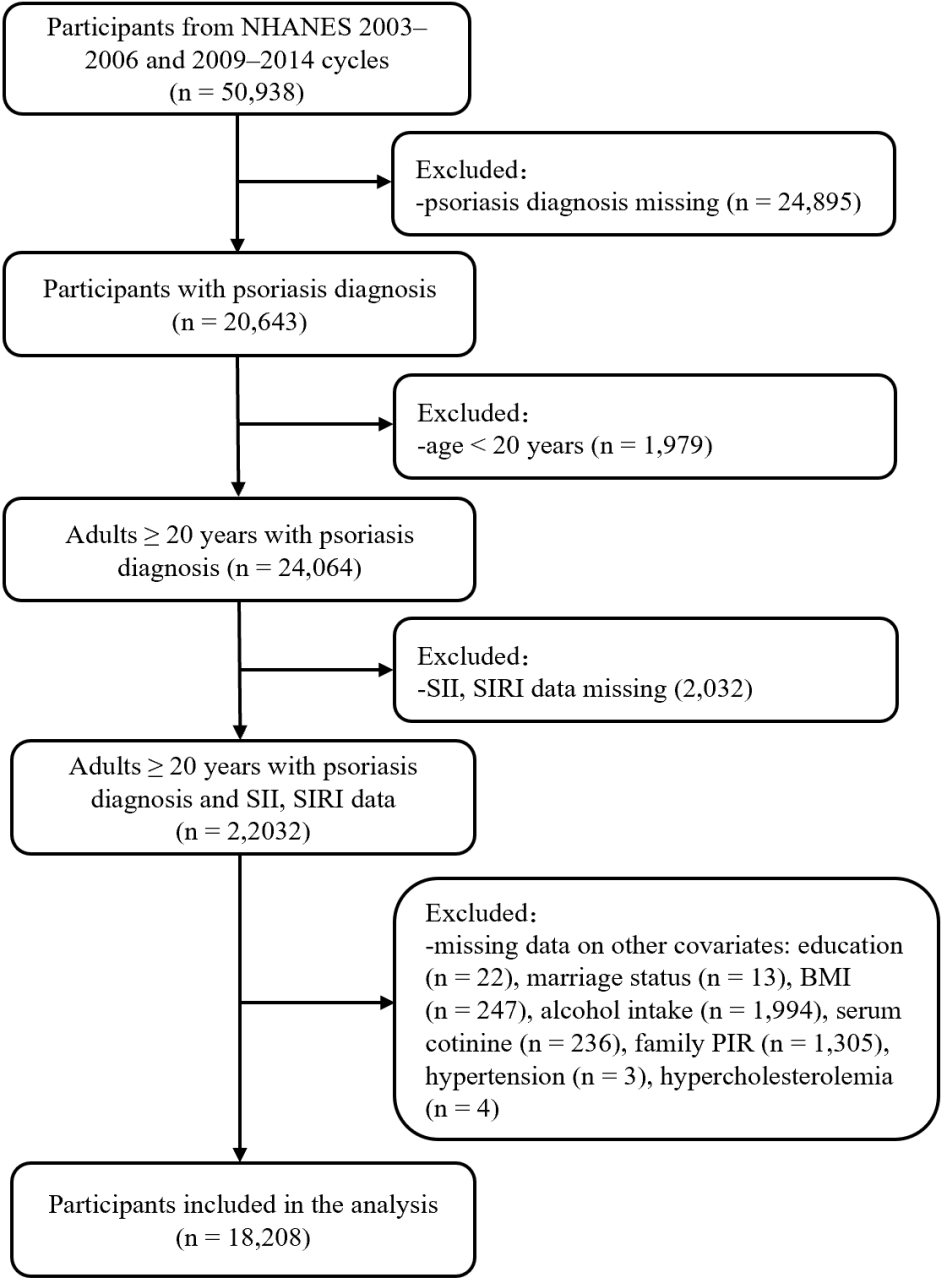
Flow chart of participant selection. BMI, body mass index; NHANES, National Health and Nutrition Examination Survey; PIR, poverty income ratio; SII, systemic immune inflammation index; SIRI, systemic inflammation response index.

This study was conducted basing on the NHANES database (11). All NHANES protocols were approved by the NCHS Research Ethics Review Board (Protocol #98-12, Continuation of Protocol #2005-06, Continuation of Protocol #2011-17, http://www.cdc.gov/nchs/nhanes/irba98.htm), and informed consent was obtained from all participants when they were enrolled. A total of 50938 participants from five NHANES cycles (2003–2004, 2005–2006, 2009–2010, 2011–2012, and 2013–2014) were enrolled in the present study. Exclusion criteria included: 1) missing psoriasis diagnosis data, 2) age <20 years old (psoriasis data for participants <20 years of age were missing in the 2003–2004 and 2005–2006 cycles), 3) missing SII or SIRI data, and 4) missing data on other covariates. A total of 18208 participants were included for analysis (**Figure 1**).

### Assessment of psoriasis

Psoriasis was defined if the participants responded affirmatively to the question, “Have you ever been told by a health care provider that you had psoriasis?” or “Have you ever been told by a doctor or other health care professional that you had psoriasis (sore-eye-asis)?” (12). Participants who refused to answer or did not know were excluded (13).

### Definition of systemic immune-inflammation index and systemic inflammation response index

Peripheral blood samples of the NHANES participants were analyzed at the Mobile Examination Centers (MEC) using a Beckman Coulter HMX Hematology Analyzer. Lymphocyte, neutrophil, monocyte, and platelet counts were measured via complete blood count, and were presented as ×10^3^ cells/μL. The SII and SIRI levels were calculated using the following formulas: platelet count × neutrophil count/lymphocyte count, monocyte count × neutrophil count/lymphocyte count, respectively. These values were expressed as ×10^3^ cells/μL based on previous studies (4, 14, 15). SII and SIRI were considered as exposure variables in this study.

### Covariates

Based on existing literature, potential confounders that may affect psoriasis were evaluated. These variables included age (20-60, >60 years old), gender (male and female), race/ethnicity (Mexican American, other Hispanic, non-Hispanic White, non-Hispanic Black, other race/multiracial), education level (high school or lower, college or above), marital status (married/living with partner, widowed/divorced/separated, never married), family poverty income ratio (PIR), alcohol intake (mild, <1 time per week; moderate, 1-3 times per week; severe, ≥4 times per week), body mass index (BMI) (<25, 25–29.9, ≥30 kg/m^2^), serum cotinine (ng/mL), hypertension (yes/no), type 2 diabetes (T2D) (yes/no), and hypercholesterolemia (yes/no). Hypertension was diagnosed if at least one of the following criteria were met: systolic pressure/diastolic pressure ≥140/90 mmHg, self-reported physician diagnosis of hypertension, or self-reported use of hypertension medication (16). T2D was diagnosed if at least one of the following criteria were met: glycosylated hemoglobin ≥6.5%, fasting blood glucose ≥7.0 mmol/L (126 mg/dL), self-reported physician diagnosis of diabetes, or self-reported use of insulin (16). Hypercholesterolemia was diagnosed if at least one of the following criteria were met: cholesterol ≥240 mg/dL, self-reported physician diagnosis of, or self-reported use of hypercholesterolemia medication (17).

### Statistical analysis

Results including geometric mean (GM), standard error (SE), percentiles, odds ratio (OR), and 95% confidence interval (CI), were adjusted using specific sample weights, clustering, and stratification to account for the complex survey design of NHANES and to ensure data representativeness of the noninstitutionalized U.S. population. And based on the NHANES analytic guidelines (18), the formula of calculating sampling weight was as follows: fasting subsample 10-year MEC weight = fasting subsample 2-year MEC weight/5 (12).

Various statistical methods were employed for data analysis. GM and SEs were reported for non-normally distributed continuous variables, while categorical variables were described in terms of frequency and percentage. Baseline characteristics between different groups were compared using the Kruskal–Wallis H test and Rao-Scott chi-square test, as appropriate. In order to categorize participants into different clusters based on their SII and SIRI measurements, the SII and SIRI data were first scaled and the k-means method was then applied. The k-means algorithm is a non-model-based technique used for categorizing mixed data (11, 19). It creates clusters in such a way that the squared Euclidean distance between the row vector of any object and the centroid vector of its corresponding cluster is minimized compared to the distances to the centroids of other clusters (20). The optimal number of clusters in this study was determined using the elbow method (20), and the subgroups were reduced in dimensionality and visualized using t-Distributed Stochastic Neighbor Embedding (t-SNE). Besides, categorical analysis on SII or SIRI levels individually was also performed by categorizing participants into three groups based on the quintile of SII/SIRI levels, including low group (1^st^ – 3^rd^ quintiles), medium group (4^th^ quintile) and high group (5^th^ quintile), respectively. Multivariate logistic regression models were used to calculate ORs and CIs to assess the associations between SII-SIRI pattern (by k-means algorithm)/SII/SIRI levels and psoriasis. For the right-skewed distribution of SII levels, SII levels were natural log-transformed (lnSII) when assessing the association between SII levels (continuous variable) and psoriasis risk. The crude model was adjusted for no covariates, while the fully adjusted model was adjusted for gender, age, race/ethnicity, education levels, marital status, BMI, alcohol intake, serum cotinine, family PIR, hypertension, T2D, and hypercholesterolemia. Additionally, four-knot (5th, 35th, 65th, and 95th quantiles) restricted cubic splines (RCS) were used to estimate exposure–response curves of SII/SIRI levels and psoriasis. A *p*-value <0.05 for overall and nonlinear indicated a non-linear relationship between SII/SIRI levels and psoriasis.

Finally, stratification analyses by gender (male, female), age (20–60, >60 years old), BMI (<25, 25–29.9, ≥30 kg/m^2^), hypertension (no and yes), T2D (no and yes), and hypercholesterolemia (no and yes) were performed, as well as interaction analyses between various stratification factors and lnSII or SIRI.

All analyses were conducted using SAS 9.4 software (SAS Institute Inc., Cary, NC, USA) and R (version 4.3.1, R Development Core Team). The R package “rms” was used for RCS analysis. A two-sided *p*-value<0.05 was considered statistically significant.

## Results

### Participants characteristics

Among 18,208 adults aged 20–80 years from five NHANES cycles, 511 (2.81%) were diagnosed with psoriasis. The baseline characteristics of the study participants, both those with and without psoriasis, are shown in **Table 1**. In brief, there were obvious differences between participants with psoriasis and those without psoriasis, including race/ethnicity, BMI, the incidence of hypertension and hypercholesterolemia, SII levels, SIRI levels, lymphocyte and neutrophil counts.

**Table 1:** Baseline characteristics of participants in the NHANES follow-up study from 2003–2006 and 2009–2014 cycles (n = 18,208).

Characteristics	Participants			*p*-value
	Total	Without psoriasis	With psoriasis	
N	18208	17697	511	
Sex				0.797
Male	8909 (49.38)	8665 (49.40)	244 (48.81)	
Female	9299 (50.62)	9032 (50.60)	267 (51.19)	
Age				0.216
20-60	13957 (82.53)	13586 (82.60)	371 (80.34)	
>60	4251 (17.47)	4111 (17.40)	140 (19.66)	
Race/ethnicity				<0.0001
Mexican American	2809 (8.18)	2767 (8.32)	42 (3.80)	
Other Hispanic	1403 (4.80)	1364 (4.85)	39 (3.51)	
Non-Hispanic White	8546 (70.11)	8227 (69.73)	319 (81.92)	
Non-Hispanic Black	3776 (10.59)	3710 (10.75)	66 (5.84)	
Other race/multiracial	1674 (6.31)	1629 (6.36)	45 (4.94)	
Education level				0.093
High school or lower	8219 (37.57)	8009 (37.71)	210 (33.48)	
College or above	9989 (62.43)	9688 (62.29)	301 (66.52)	
Alcohol intake (times/week)				0.216
Mild (<1)	12275 (62.05)	11932 (62.12)	343 (59.75)	
Moderate (1-3)	4204 (26.25)	4094 (26.28)	110 (25.35)	
Severe (≥)	1729 (11.70)	1671 (11.59)	58 (14.90)	
Marital status				0.565
Married/living with partner	11032 (64.49)	10729 (31.43)	303 (64.28)	
widowed/divorced/separated	3600 (16.91)	3474 (32.30)	126 (18.58)	
Never married	3576 (18.60)	3494 (36.27)	82 (17.14)	
BMI (kg/m2)				0.0006
<25	5460 (31.12)	5349 (31.43)	111 (21.48)	
25-29.9	5866 (32.40)	5694 (32.30)	172 (35.61)	
≥30	6882 (36.48)	6654 (36.27)	228 (42.92)	
Serum cotinine (ng/mL)	0.35 ± 0.03	0.36 ± 0.03	0.29 ± 0.06	0.272
Family PIR	2.42 ± 0.05	2.42 ± 0.04	2.51 ± 0.11	0.130
Hypertension (yes)	6824 (34.19)	6575 (33.81)	249 (45.88)	<0.0001
T2D (yes)	2524 (9.68)	2270 (9.63)	90 (11.20)	0.289
Hypercholesterolemia (yes)	6986 (37.76)	6734 (37.44)	252 (47.62)	<0.0001
SII (10^3^ cells/μL)	488.17 ± 3.70	486.57 ± 3.73	542.72 ± 14.23	<0.0001
SIRI (10^3^ cells/μL)	1.05 ± 0.01	1.05 ± 0.01	1.17 ± 0.04	0.0006
Lymphocyte (10^3^ cells/μL)	2.01 ± 0.01	2.01 ± 0.01	1.93 ± 0.03	0.019
Monocyte (10^3^ cells/μL)	0.52 ± 0.00	0.52 ± 0.00	0.53 ± 0.01	0.774
Neutrophil (10^3^ cells/μL)	4.04 ± 0.02	4.03 ± 0.02	4.25 ± 0.08	0.003
peripheral platelet (10^3^ cells/μL)	242.72 ± 0.95	242.55 ± 0.96	245.73 ± 3.24	0.142

Data are expressed as geometric mean ± SE or frequency (percentage). Percentages, geometric mean, and SE were weight-adjusted using NHANES-specified sampling weights. For categorical variables, p-values were calculated using Rao-Scott chi-square test, and for continuous variables, p-values were calculated using Kruskal–Wallis H test (non-normal distribution). —, not applicable; BMI, body mass index; NHANES, National Health and Nutrition Examination Survey; PIR, poverty income ratio; Q, quantile; SE, standard error; SII, systemic immune–inflammation index; SIRI, systemic inflammation response index; T2D, type 2 diabetes.

**Figure 2:**
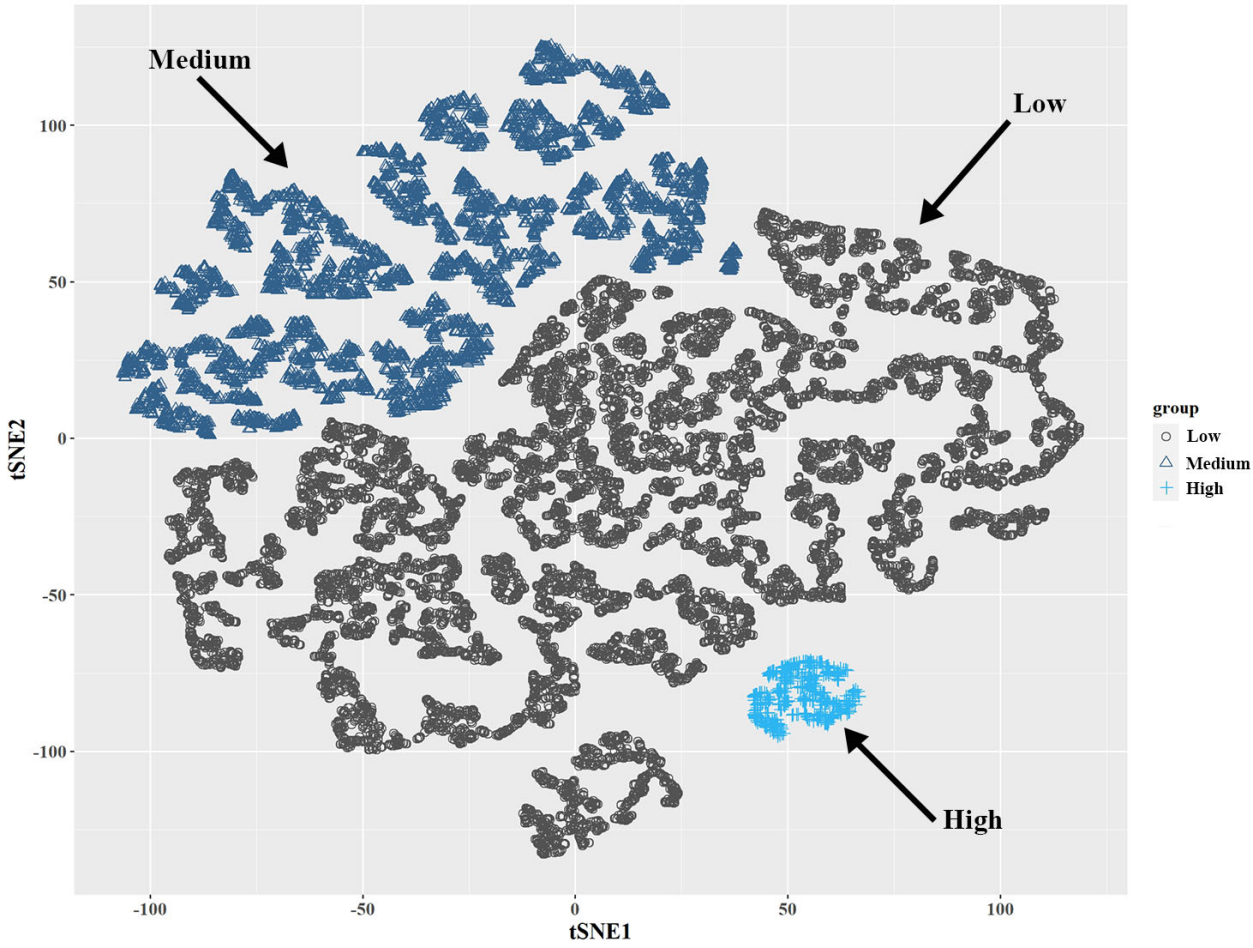
Visualization of k-means clustering using t-SNE for NHANES participants (2003-2006, 2009-2014, n = 18,208) based on SII and SIRI levels. Three sub-groups (low-, medium-, and high-level groups) were identified based on the combination of SII and SIRI levels. NHANES, National Health and Nutrition Examination Survey; SII, systemic immune–inflammation index; SIRI, systemic inflammation response index; t-SNE, t-Distributed Stochastic Neighbor Embedding.

A total of 18,208 participants were also clustered into three subgroups based on SII–SIRI pattern (by k-means algorithm, **Figure 2**), SII levels, and SIRI levels, respectively. As shown in **Table 2**, SII-SIRI pattern/SII/SIRI levels were significantly associated with gender, age, race/ethnicity, education levels, alcohol intake, marital status, serum cotinine levels, the incidence of psoriasis, hypertension, T2D and hypercholesterolemia. And the levels of SII, SIRI, lymphocyte, monocyte, neutrophil and peripheral platelet counts were significantly different among the three subgroups (**Table 3**). Comparing to low–level group, the high–level group had the highest levels of SII, SIRI, monocyte, neutrophil and peripheral platelet counts, and the lowest lymphocyte counts.

**Table 2:** Baseline characteristics of participants based on subgroups of SII-SIRI pattern/SII/SIRI levels in the NHANES follow-up study from 2003–2006 and 2009–2014 cycles (n = 18,208).

Characteristics	SII-SIRI pattern divided by k-means method				SII levels (10^3^ cells/μL)				SIRI levels (10^3^ cells/μL)			
	Low	Medium	High	*p*-value	Low (Q1-Q3, <549.50)	Medium (Q4, 549.50-737.69)	High (Q5, ≥737.69)	*p*-value	Low (Q1-Q3, <1.18)	Medium (Q4, 1.18-1.65)	High (Q5, ≥1.65)	*p*-value
N	12606	5130	472		11103	3492	3613		11032	3458	3718	
SII-SIRI pattern								<0.0001				<0.0001
Low	–	–	–	–	10591 (95.50)	1816 (52.78)	199 (5.24)		10661 (96.47)	1859 (53.26)	86 (1.95)	
Medium	–	–	–	–	510 (4.48)	1673 (47.17)	2947 (82.71)		371 (3.53)	1595 (46.60)	3164 (86.08)	
High	–	–	–	–	2 (0.02)	3 (0.06)	467 (12.04)		0 (0.00)	4 (0.14)	468 (11.97)	
Sex				0.134				<0.0001				<0.0001
Male	6209 (49.42)	2456 (48.80)	244 (55.00)		5834 (52.85)	1632 (47.49)	1443 (40.87)		5137 (46.40)	1779 (52.09)	1993 (55.63)	
Female	6397 (50.58)	2674 (51.20)	228 (45.00)		5269 (47.15)	1860 (52.51)	2170 (59.13)		5895 (53.60)	1679 (47.91)	1725 (44.37)	
Age				<0.0001				0.909				<0.0001
20-60	9831 (83.98)	3805 (79.87)	321 (72.82)		8451 (82.50)	2688 (82.79)	2818 (82.35)		8777 (85.23)	2598 (81.59)	2582 (75.38)	
>60	2775 (16.03)	1325 (20.13)	151 (27.18)		2652 (17.50)	804 (17.21)	795 (17.65)		2255 (14.77)	860 (18.41)	1136 (24.62)	
Race/ethnicity				<0.0001				<0.0001				<0.0001
Mexican American	1979 (8.59)	772 (7.39)	58 (5.81)		1654 (8.22)	568 (8.32)	587 (7.91)		1711 (8.54)	578 (8.21)	520 (7.06)	
Other Hispanic	1018 (5.12)	358 (4.18)	27 (3.22)		880 (4.93)	276 (5.03)	247 (4.20)		880 (5.11)	2665 (4.77)	258 (3.93)	
Non-Hispanic White	5355 (67.29)	2886 (75.95)	305 (81.44)		4757 (67.59)	1822 (73.40)	1967 (74.40)		4491 (66.11)	1838 (74.21)	2217 (78.06)	
Non-Hispanic Black	3017 (12.37)	699 (6.68)	60 (6.41)		2733 (12.72)	545 (7.78)	498 (7.02)		2825 (13.39)	486 (6.65)	465 (6.13)	
Other race/multiracial	1237 (6.64)	415 (5.80)	22 (3.12)		1079 (6.54)	281 (5.47)	314 (6.46)		1125 (6.86)	291 (6.16)	258 (4.82)	
Education level				0.0002				0.049				<0.0001
High school or lower	5610 (36.56)	2370 (39.57)	239 (42.84)		4972 (36.88)	1570 (37.49)	1677 (39.76)		4816 (35.87)	1620 (38.79)	1783 (41.48)	
College or above	6996 (63.44)	2760 (60.43)	233 (57.16)		6131 (63.12)	1992 (62.51)	1936 (60.24)		6216 (64.13)	1838 (61.21)	1935 (58.52)	
Alcohol intake (times/week)				0.013				<0.0001				0.004
Mild (<1)	8470 (61.51)	3490 (63.31)	315 (62.37)		7339 (60.15)	2400 (64.37)	2536 (65.44)		7453 (61.72)	2322 (62.83)	2500 (62.28)	
Moderate (1-3)	2981 (27.15)	1124 (24.33)	99 (23.49)		2700 (28.11)	763 (23.90)	741 (23.02)		2610 (27.30)	796 (25.45)	798 (23.93)	
Severe (≥)	1155 (11.34)	516 (12.35)	58 (14.14)		1064 (11.74)	329 (11.73)	336 (11.54)		969 (10.99)	340 (11.72)	420 (13.80)	
Marital status				<0.0001				<0.0001				<0.0001
Married/living with partner	7708 (65.64)	3049 (62.12)	275 (59.68)		6780 (65.74)	2141 (64.76)	2111 (60.45)		6696 (65.22)	2134 (65.31)	2202 (61.52)	
widowed/divorced/separated	2385 (15.56)	1102 (19.70)	113 (22.25)		2119 (15.53)	691 (17.42)	790 (20.55)		2088 (15.78)	672 (16.66)	840 (20.54)	
Never married	2513 (18.79)	979 (18.18)	84 (18.07)		2204 (18.73)	660 (17.81)	712 (19.00)		2248 (19.00)	652 (18.04)	676 (17.94)	
BMI (kg/m^2^)				<0.0001				<0.0001				<0.0001
<25	3921 (32.51)	1385 (27.39)	154 (35.86)		3455 (32.41)	1020 (29.78)	985 (28.59)		3497 (33.37)	946 (27.80)	1017 (27.68)	
25-29.9	4105 (32.91)	1612 (31.51)	149 (28.51)		3699 (33.75)	1086 (31.86)	1081 (28.89)		3542 (32.46)	1116 (33.04)	1208 (31.60)	
≥30	4580 (34.58)	2133 (41.09)	169 (35.63)		3949 (33.83)	1386 (38.36)	1547 (42.51)		3993 (34.17)	1396 (39.16)	1493 (40.72)	
Serum cotinine (ng/mL)	0.30 ± 0.02	0.48 ± 0.05	0.91 ± 0.20	<0.0001	0.32 ± 0.02	0.36 ± 0.04	0.48 ± 0.05	0.0003	0.28 ± 0.02	0.39 ± 0.05	0.62 ± 0.07	<0.0001
Family PIR	2.44 ± 0.05	2.39 ± 0.05	2.36 ± 0.11	0.237	2.44 ± 0.05	2.42 ± 0.05	2.35 ± 0.06	0.166	2.45 ± 0.04	2.39 ± 0.06	2.36 ± 0.06	0.134
Psoriasis (yes)	299 (2.70)	192 (4.10)	20 (4.11)	0.001	271 (2.63)	104 (3.57)	136 (4.24)	0.0007	255 (2.68)	107 (3.36)	149 (4.29)	0.0017
Hypertension (yes)	4513 (31.64)	2099 (39.71)	212 (41.43)	<0.0001	4066 (32.45)	1354 (35.18)	1404 (38.41)	<0.0001	3831 (30.48)	1366 (37.35)	1627 (42.16)	<0.0001
T2D (yes)	1632 (8.55)	811 (12.19)	81 (12.09)	<0.0001	1496 (8.83)	454 (9.34)	574 (12.54)	<0.0001	1361 (7.94)	528 (11.10)	625 (13.47)	<0.0001
Hypercholesterolemia (yes)	4718 (36.92)	2076 (39.80)	192 (37.65)	0.019	4217 (37.55)	1318 (36.91)	1451 (39.26)	0.278	4043 (36.21)	1416 (40.43)	1527 (39.76)	0.0001

Data are expressed as GM ± SE or frequency (percentage). Percentages, geometric mean, SE and cut points were weight-adjusted using NHANES-specified sampling weights. For categorical variables, p-values were calculated using Rao-Scott chi-square test, and for continuous variables, p-values were calculated using Kruskal–Wallis H test (non-normal distribution). —, not applicable; BMI, body mass index; GM, geometric mean; NHANES, National Health and Nutrition Examination Survey; PIR, poverty income ratio; Q, quantile; SE, standard error; SII, systemic immune–inflammation index; SIRI, systemic inflammation response index; T2D, type 2 diabetes.

**Table 3:** Comparison of SII, SIRI levels and whole blood lymphocyte, monocyte, neutrophil and peripheral platelet counts of the NHANES participants (2003–2006 and 2009–2014 cycles, n = 18,208) among subgroups of SII-SIRI pattern/SII/SIRI levels (10^3^ cells/μL).

Characteristics	SII-SIRI pattern divided by k-means method				SII levels (10^3^ cells/μL)				SIRI levels (10^3^ cells/μL)			
	Low	Medium	High	*p*-value	Low (Q1-Q3, <549.50)	Medium (Q4, 549.50-737.69)	High (Q5, ≥737.69)	*p*-value	Low (Q1-Q3, <1.18)	Medium (Q4, 1.18-1.65)	High (Q5, ≥1.65)	*p*-value
N	12606	5130	472		11103	3492	3613		11032	3458	3718	
SII	384.48 ± 2.23	784.92 ± 5.33	1565.58 ± 32.32	<0.0001	352.91 ± 1.73	631.83 ± 1.21	997.97 ± 5.57	<0.0001	384.11 ± 2.89	585.59 ± 5.09	834.97 ± 8.09	<0.0001
SIRI	0.79 ± 0.00	1.81 ± 0.01	4.35 ± 0.06	<0.0001	0.80 ± 0.01	1.29 ± 0.01	1.92 ± 0.02	<0.0001	0.73 ± 0.00	1.38 ± 0.00	2.37 ± 0.02	<0.0001
Lymphocyte	2.11 ± 0.01	1.83 ± 0.01	1.40 ± 0.03	<0.0001	2.14 ± 0.01	1.92 ± 0.02	1.73 ± 0.01	<0.0001	2.10 ± 0.01	1.99 ± 0.01	1.77 ± 0.01	<0.0001
Monocyte	0.48 ± 0.00	0.61 ± 0.00	0.76 ± 0.02	<0.0001	0.50 ± 0.00	0.53 ± 0.00	0.56 ± 0.01	<0.0001	0.45 ± 0.00	0.59 ± 0.00	0.70 ± 0.01	<0.0001
Neutrophil	3.47 ± 0.02	5.46 ± 0.03	8.00 ± 0.16	<0.0001	3.39 ± 0.02	4.65 ± 0.03	5.92 ± 0.04	<0.0001	3.37 ± 0.02	4.70 ± 0.03	5.96 ± 0.04	<0.0001
peripheral platelet	233.56 ± 0.85	263.57 ± 1.52	273.17 ± 4.87	<0.0001	222.91 ± 0.78	260.97 ± 1.40	291.40 ± 1.50	<0.0001	239.21 ± 1.01	248.28 ± 1.67	247.89 ± 1.51	<0.0001

Data are expressed as GM ± SE. GM and SE were weight-adjusted using NHANES-specified sampling weights. p-values were calculated using Kruskal–Wallis H test. GM, geometric mean; NHANES, National Health and Nutrition Examination Survey; SE, standard error; SII, systemic immune–inflammation index; SIRI, systemic inflammation response index.

### Association between SII-SIRI pattern/SII/SIRI levels and the risk of psoriasis

As presented in **Table 4**, medium levels of SII-SIRI pattern were associated with an increased risk of psoriasis (medium vs. low, OR = 1.54, 95% CI: 1.20, 1.98, *p*-trend = 0.0002) in the crude model. The results remained robust and statistically significant (medium vs. low, OR = 1.40, 95% CI: 1.09, 1.81, *p*-trend = 0.0031) after multivariable adjustment. Compared to the low–level group of SII, multivariate-adjusted OR for participants in the high–level group tend to be higher (high vs. low, OR = 1.52, 95% CI: 1.18, 1.95, *p*-trend = 0.0014). Similar results were found for SIRI levels (high vs. low, OR = 1.48, 95% CI: 1.12, 1.95, *p*-trend = 0.007). When lnSII or SIRI levels were included as continuous variables in the multivariate logistic regression models, the positive associations still existed (OR = 1.39, 95% CI: 1.12, 1.74, *p* = 0.0038; OR = 1.10, 95% CI: 1.02, 1.18, *p* = 0.016, respectively).

**Table 4:** Odds ratios (95%CI) of psoriasis and SII-SIRI pattern/SII/SIRI levels in the NHANES follow-up study from 2003–2006 and 2009–2014 cycles (n = 18,208) (10^3^ cells/μL).

Categories	Psoriasis incidence		
	psoriasis/observations (n/N)	Unadjusted OR (95% CI)	Adjusted OR (95% CI)
SII-SIRI pattern			
Low	299 (12307)	Ref.	Ref.
Medium	192 (5130)	1.54 (1.20, 1.98)^***^	1.40 (1.09, 1.81) ^*^
High	20 (472)	1.54 (0.83, 2.88)	1.46 (0.79, 2.70)
*p*-value for trend	–	0.0002	0.0031
SII levels			
Low (Q1-Q3, <549.50)	271 (11103)	Ref.	Ref.
Medium (Q4, 549.50-737.69)	104 (3492)	1.37 (1.01, 1.86)^*^	1.30 (0.96, 1.78)
High (Q5, ≥737.69)	136 (3613)	1.64 (1.27, 2.12)^***^	1.52 (1.18, 1.95)^**^
*p*-value for trend	–	0.0002	0.0014
Continuous (lnSII)	–	1.51 (1.22, 1.87)^***^	1.39 (1.12, 1.74)^**^
SIRI levels			
Low (Q1-Q3, <1.18)	255 (11032)	Ref.	Ref.
Medium (Q4, 1.18-1.65)	107 (3458)	1.27 (0.95, 1.68)	1.16 (0.86, 1.55)
High (Q5, ≥1.65)	149 (3718)	1.63 (1.23, 2.15)^***^	1.48 (1.12, 1.95)^**^
*p*-value for trend	–	0.0005	0.007
Continuous	–	1.14 (1.07, 1.22)^***^	1.10 (1.02, 1.18)^*^

ORs were estimated using multivariate logistic regression models and were weight adjusted using NHANES-specified sampling weights. ORs were adjusted for gender, age, race/ethnicity, education levels, marital status, BMI, alcohol intake, serum cotinine, family PIR, hypertension, type 2 diabetes, and hypercholesterolemia. ^*^p<0.05, ^**^p<0.01, ^***^p<0.001. BMI, body mass index; CI, confidence interval; NHANES, National Health and Nutrition Examination Survey; OR, odds ratio; PIR, poverty income ratio; Ref, reference; SII, systemic immune–inflammation index; SIRI, systemic inflammation response index.

Based on the association between the SII-SIRI pattern and psoriasis, we aimed to determine the range of SII and SIRI levels that were positively associated with an increased risk of psoriasis. As shown in **Table 5**, when SII levels were higher than 737.69 × 10^3^ cells/μL, and SIRI levels ranged from 1.18 to 1.65 × 10^3^ cells/μL, the risk of psoriasis increased significantly (OR = 2.17, 95% CI: 1.25, 3.79, *p* = 0.007). No significant associations were found among other concentration ranges.

**Table 5:** Multivariable-adjusted OR (95% CI) of psoriasis according to subgroups of SII levels stratified by SIRI levels (10^3^ cells/μL).

	SII levels				*p*-value for trend
	geometric mean ± SD	Low (Q1-Q3, <549.50)	Medium (Q4, 549.50-737.69)	High (Q5, ≥737.69)	
SIRI levels					
Low (Q1-Q3, <1.18)	384.14 ± 2.90	Ref.	1.14 (0.72, 1.83)	1.16 (0.59, 2.29)	0.540
Medium (Q4, 1.18-1.65)	585.59 ± 5.09	Ref.	1.32 (0.74, 2.34)	2.17 (1.25, 3.79)^**^	0.008
High (Q5, ≥1.65)	834.97 ± 8.09	Ref.	1.26 (0.59, 2.67)	1.07 (0.60, 1.92)	0.910

ORs were estimated using multivariate logistic regression models and were weight adjusted using NHANES-specified sampling weights. ORs were adjusted for gender, age, race/ethnicity, education levels, marital status, BMI, alcohol intake, serum cotinine, family PIR, hypertension, type 2 diabetes, and hypercholesterolemia. ^**^p<0.01. BMI, body mass index; CI, confidence interval; NHANES, National Health and Nutrition Examination Survey; OR, odds ratio; PIR, poverty income ratio; Ref, reference; SII, systemic immune–inflammation index; SIRI, systemic inflammation response index.

### Dose-response relationship between SII/SIRI levels and the risk of psoriasis

**Figure 3:**
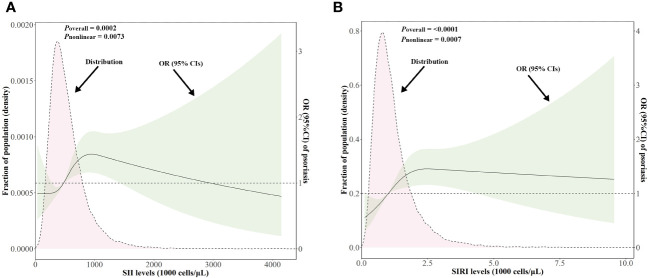
Distributions of SII and SIRI levels (10^3^ cells/μL) and dose-response curves of SII and SIRI levels in relation to psoriasis in the NHANES follow-up study from 2003–2006 and 2009–2014 cycles (n = 18,208). Distributions of SII and SIRI levels and adjusted ORs with 95% CIs for **(A)** SII levels, **(B)** SIRI levels. ORs for SII/SIRI levels were adjusted for gender, age, race/ethnicity, education levels, marital status, BMI, alcohol intake, serum cotinine, family PIR, hypertension, type 2 diabetes, and hypercholesterolemia. BMI, body mass index; CI, confidence interval; NHANES, National Health and Nutrition Examination Survey; OR, odds ratio; PIR, poverty income ratio; SII, systemic immune–inflammation index; SIRI, systemic inflammation response index.

As shown in **Figure 3**, after adjusting for multiple potential confounders, the nonlinear associations between SII/SIRI levels and the risk of psoriasis were statistically significant (*p* value for overall < 0.05 and *p* value for nonlinear < 0.05). As SII increased from 486.75 to 1418.09 × 10^3^ cells/μL, the OR (95% CI) of psoriasis increased from 1.02 (1.01, 1.02) to 1.33 (1.00, 1.77), indicating a positive association between the SII levels and psoriasis risk within this range. Similarly, a positive association existed between SIRI levels and psoriasis risk (from 1.03 (1.01, 1.04) to 1.40 (1.00, 1.96)) when SIRI ranged from 1.06 to 4.29 × 10^3^ cells/μL.

### Subgroup analysis

**Figure 4:**
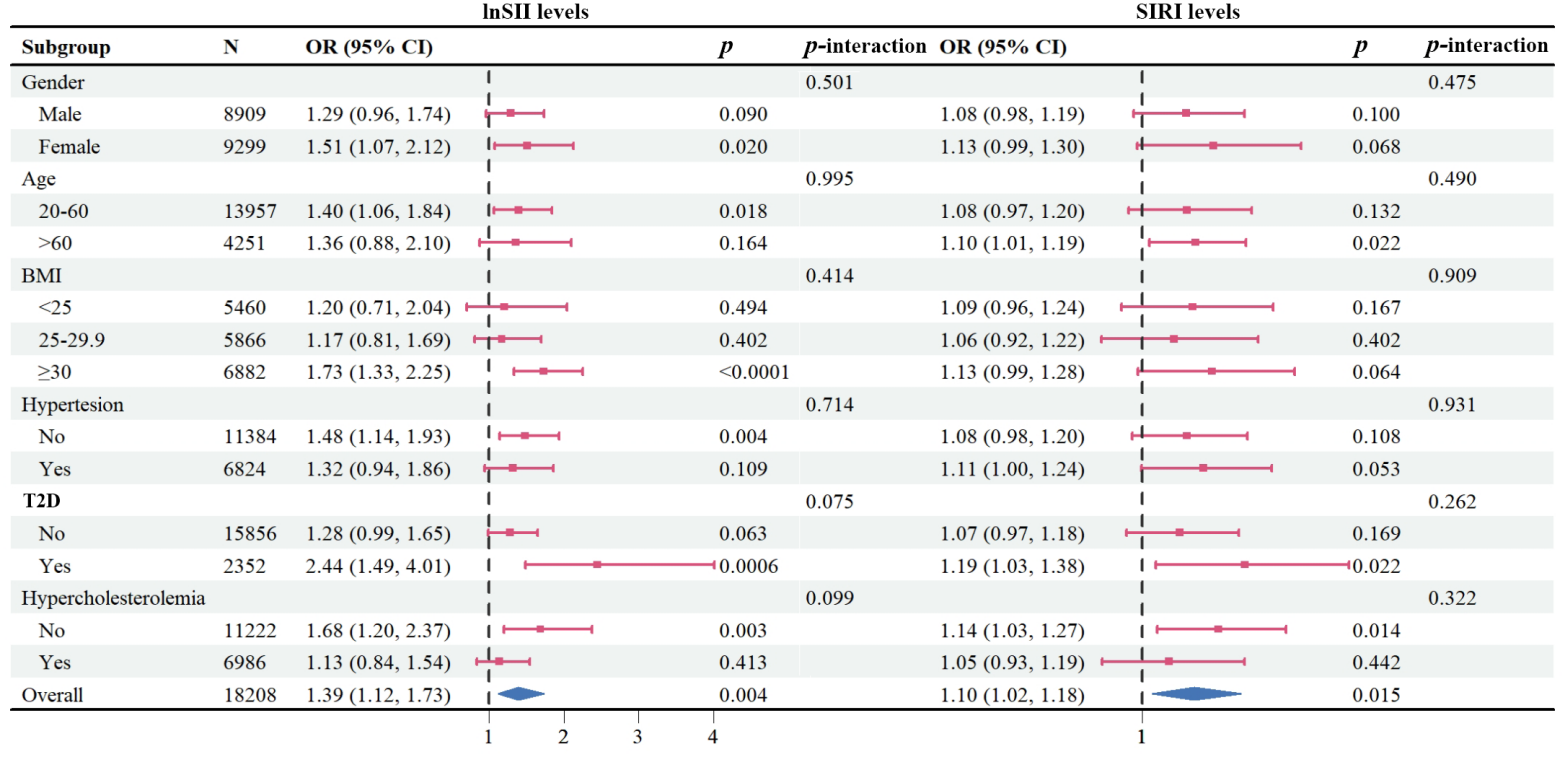
Forest plot depicting subgroup analysis of the association between lnSII/SIRI and psoriasis. The ORs were calculated using multivariate logistic regression models with adjustment for gender, age, race/ethnicity, education levels, marital status, BMI, alcohol intake, serum cotinine, family PIR, hypertension, type 2 diabetes, and hypercholesterolemia, except for the variable used for stratification. BMI, body mass index; ORs, Odds ratios; PIR, poverty income ratio; SII, systemic immune–inflammation index; SIRI, systemic inflammation response index.

To further study the roles of potential confounders in the associations of SII/SIRI levels with psoriasis, we divided the participants in subgroups stratified by gender, age, BMI, hypertension, T2D, and hypercholesterolemia (**Figure 4**). In subgroups analysis of SII levels, a statistically significant association was only observed in females, in the 20-60 years age group, in those with a BMI ≥ 30 kg/m2, without hypertension, with T2D, or without hypercholesterolemia (all *p* < 0.05). In the subgroup analysis of SIRI levels, positive associations were found in older adults (> 60 years of age), those with T2D, or without hypercholesterolemia (all *p* < 0.05). However, we did not find any significant interactions between SII/SIRI levels and those potential confounders (all *p* value for interaction > 0.05).

## Discussion

To our knowledge, this current cross-sectional study is the first to investigate the association between the SII-SIRI pattern, as well as SII and SIRI individually, and the risk of psoriasis in a large, nationally representative sample. The results of this study revealed that significant changes in the SII-SIRI pattern are independently associated with an increased risk of psoriasis in the NHANES population. Interestingly, the association between SII or SIRI levels and the occurrence of psoriasis exhibited a non-linear dose-response relationship. Additionally, our findings suggest that monitoring SII and SIRI levels and combining these two indexes in analysis may assist in the early identification of individuals at high risk of developing psoriasis. Furthermore, prioritizing the management of inflammation may be worth considering in order to mitigate the risk of psoriasis.

Psoriasis is a chronic inflammatory skin disease characterized by abnormal innate and acquired immunity (1). The presence of an abundance of neutrophils in the skin lesions of psoriasis is a typical histopathological hallmark (21), and their release of cytokines, chemokines, enzymes, and neutrophil elastase mediates chronic inflammation (22). Monocytes play a central role in innate immune system and have a significant function in orchestrating inflammation (23). Lymphocytes are key component cells for adaptive immune responses, which links the innate and adaptive responses (24). Platelets maintain homeostasis, participate in mediating acute and chronic inflammatory processes, and contribute to the creation of an inflammatory environment (25). Previous studies have shown that changes in neutrophils, monocytes, lymphocytes, and platelet cells from peripheral blood are associated with psoriasis (26–28). SII and SIRI have been reported as promising systemic inflammatory response biomarkers in predicting stroke prognosis, colorectal cancer, gynecological and breast cancers (29–32). SII has also been found to be associated with the psoriatic comorbidities including hypertension, T2D, hyperlipidemia, nonalcoholic fatty liver disease, and psoriatic arthritis (16, 33–36). However, previous literature on the association between SII and psoriasis is limited to two small sample size cross-sectional studies that examined the predictive ability and disease severity (37, 38). Thus, we hypothesized that SII or SIRI may be associated with the occurrence of psoriasis.

In our study, we found that patients with psoriasis had significantly higher SII and SIRI levels compared to those without psoriasis. In addition to considering SII or SIRI as single exposure variables, we also conducted an unsupervised clustering model to group the SII-SIRI mixture as a pattern and studied the association between this pattern and the risk of psoriasis. We found that higher levels of SII or SIRI were associated with an increased risk of psoriasis. Besides, the medium level of the SII-SIRI pattern was positively associated with psoriasis, specifically when SII levels were higher than 737.69 × 10^3^ cells/μL and SIRI levels ranged from 1.18 to 1.65 × 10^3^ cells/μL. This concentration range had the highest risk of psoriasis compared to considering SII or SIRI levels individually, suggesting that the SII-SIRI pattern provides more clinical information than a single index. Interestingly, the results of the restricted cubic spline analysis demonstrated a non-linear association between SII/SIRI levels and psoriasis. It is worth noting that previous SII-related studies have reported a non-linear dose-response relationship between SII and hyperlipidemia, all-cause mortality in patients with nonalcoholic fatty liver disease, and a ‘U-shaped’ association with all-cause, cardiovascular disease, and cancer-related mortality in cardiovascular disease patients (16, 35, 39). These findings indicate that SII or SIRI levels and psoriasis occurrence is intricate and dose-dependent, which worths further studies.

In subgroup analysis, we found that the positive associations between SII and psoriasis were present in females, people aged younger than 60, obese people, and those with T2D. Similarly, positive associations were observed between SIRI and psoriasis in people aged older than 60 and those with T2D. These findings suggest that the association between SII or SIRI and psoriasis occurrence may be influenced by other confounding factors, and obesity and T2D may be risk factors for psoriasis, as previously reported by numerous studies (40–42). Similar results could be seen from the epidemiological study between SII and kidney stone (10). Further interventional/experimental research is needed to explore the potential underlying mechanisms behind these findings.

Our study has several notable advantages. Firstly, the large sample size and appropriate adjustment of covariates support the reliability and representativeness of our study. Secondly, we thoroughly assessed the individual effects of SII or SIRI on psoriasis risk, addressing previous research gaps. Furthermore, we used a SII-SIRI pattern grouping method to investigate the relationship between the SII-SIRI mixture pattern and psoriasis risk using different statistical models, obtaining relatively robust and consistent results, which increases the reliability of our study. And we found the changing threshold or ranges of SII and SIRI concentrations, which were associated with the risk of psoriasis most significantly than taking SII or SIRI into consideration individually. This finding might be hoping to support the early identification and prevention of psoriasis. Lastly, SII and SIRI were measured using common methodology, making them easily accessible and low-cost biomarkers with potential clinical utility. However, there are a few limitations worth noting. Firstly, due to the cross-sectional study design, we cannot establish a causal association between the SII-SIRI pattern/SII/SIRI and psoriasis risk. Secondly, the diagnosis of psoriasis was based on self-reported questionnaires, introducing the possibility of recall bias. Lastly, although we adjusted for a set of confounders, there may still be residual or unmeasured confounders in our findings. Therefore, it is crucial to confirm the association between the SII-SIRI pattern/SII/SIRI and psoriasis risk in future prospective studies with larger sample sizes and more comprehensive data collection.

## Conclusion

Our cross-sectional study provides evidence that SII or SIRI is positively associated with the risk of psoriasis. Additionally, we established a novel SII-SIRI pattern and observed a similar association when SII and SIRI levels fall within a specific threshold range. However, given the limitations of our study, further research with well-designed prospective designs is needed to confirm these findings.

## Data availability statement

The original contributions presented in the study are included in the article/supplementary materials, further inquiries can be directed to the corresponding author/s.

## Ethics statement

The studies involving humans were approved by Ethics Review Committee of the National Center for Health Statistics. The studies were conducted in accordance with the local legislation and institutional requirements. The participants provided their written informed consent to participate in this study.

## Author contributions

RM: Conceptualization, Formal analysis, Funding acquisition, Investigation, Methodology, Software, Writing – original draft. LC: Conceptualization, Formal analysis, Investigation, Methodology, Software, Visualization, Writing – original draft. JC: Conceptualization, Formal analysis, Investigation, Methodology, Software, Validation, Writing – original draft. NY: Data curation, Resources, Writing – original draft. YW: Data curation, Resources, Writing – original draft. QC: Data curation, Resources, Writing – original draft. WC: Data curation, Funding acquisition, Writing – original draft. CP: Data curation, Funding acquisition, Writing – original draft. HQ: Data curation, Funding acquisition, Writing – original draft. YD: Data curation, Resources, Writing – original draft. XW: Conceptualization, Funding acquisition, Investigation, Visualization, Writing – original draft. QY: Funding acquisition, Resources, Validation, Writing – review & editing. YS: Conceptualization, Formal analysis, Funding acquisition, Resources, Validation, Writing – review & editing.
